# Experimental accelerating testicular tissue recovery post-methotrexate treatment in rats: A promising role of Sertoli cell-conditioned medium: An experimental study

**DOI:** 10.18502/ijrm.v22i4.16390

**Published:** 2024-06-12

**Authors:** Aref Delkhosh, Fatemeh Shabani, Masoud Delashoub

**Affiliations:** ^1^Department of Pathobiology, Faculty of Veterinary Medicine, Division of Pathology, Urmia University, Urmia, Iran.; ^2^Midwifery Department, Faculty of Nursing and Midwifery, Tabriz University of Medical Sciences, Tabriz, Iran.; ^3^Department of Basic Sciences, Faculty of Veterinary Medicine, Tabriz Medical Sciences, Islamic Azad University, Tabriz, Iran.; ^4^Department of Basic Sciences, Biotechnology Research Centre, Tabriz Branch, Islamic Azad University, Tabriz, Iran.

**Keywords:** Sertoli cell, Conditioned culture media, Methotrexate, Testis, Histopathology, Rats.

## Abstract

**Background:**

Methotrexate (MET) is one of the most important chemotherapy agents used against various tumors and cancer diseases. One of the critical side effects of MET is inducing male infertility.

**Objective:**

The current study aimed to investigate Sertoli cell culture-conditioned medium (SCM) recovery effects on MET-induced conditions in rats.

**Materials and Methods:**

30 mature male Wistar rats were randomly divided into 3 groups (n = 10). In the first group, rats received normal saline intraperitoneally. In the second group, animals received MET (10 mg/kg; intraperitoneally) once a week for 2 wk. The rats in the third group (MET+SCM) received MET and a single injection of SCM for 56 days post-MET administration. 56 days later, serum, epididymis, and testicular tissue samples were collected, and the animals were euthanized. Sperm parameters, serum levels of luteinizing hormone, follicle-stimulating hormone, and testosterone were examined. The testicular tissues were stained using hematoxylin and eosin solution, and histopathological changes were analyzed.

**Results:**

The MET-induced condition resulted in significant pathological changes in the testis, decreased hormone levels, and downregulated sperm parameters. However, SCM injection improved hormonal levels, testicular changes, and sperm parameters.

**Conclusion:**

It can be concluded that a single intra-testicular SCM injection accelerates male reproductive system recovery post-MET treatment.

## 1. Introduction

Male infertility is characterized by the inability of a man to successfully impregnate a female partner despite engaging in regular unprotected sexual intercourse. On the other hand, cancer treatments, such as chemotherapy and radiation therapy, can profoundly affect male fertility by causing damage to the sperm-producing cells in the testicles (1). This damage often results in a decrease in sperm production.

Methotrexate (MET) is a chemotherapeutic agent commonly employed in the treatment of cancer. It is particularly utilized in higher doses for the management of malignant tumors, but it can also be prescribed for certain benign tumor cases. Research has demonstrated that the primary mechanism of action of MET involves the inhibition of the cell cycle machinery of cancer cells, predominantly targeting the S phase. In addition to its use in cancer treatment, MET is found to be effective in reducing inflammation and controlling the symptoms associated with this autoimmune condition, hence it can be used to treat some diseases such as rheumatoid arthritis and Crohn's disease. MET, like many other cancer drugs, is associated with certain side effects that are related to the induction of oxidative stress (OS) (2). One of the notable side effects is the arrest of oogenesis and spermatogenesis, which has been identified as a significant adverse effect. Despite MET having the potential to disrupt the testicular microenvironment, diminish the number of spermatogonial stem cells (SSCs) crucial for sperm production, and affect sperm DNA integrity (3), it is crucial to recognize that this spermatogenesis failure is a reversible condition. Ceasing the use of the drug allows for the possibility of recovering sperm production (4).

On the contrary, it has been shown that the administration of antioxidant agents in conditions induced by MET can reduce OS in testicular tissues (5). However, achieving complete protection against the adverse effects of MET treatment has not been accomplished (6). Additionally, it has been demonstrated that the conditioned medium derived from testicular cells has the ability to induce in vitro gametogenesis and support the development of germ cells from human embryonic stem cells (7). One type of testicular cell that plays a crucial role in this process is Sertoli cells. It act as nurse cells and provides essential factors, including growth factors such as glial cell line-derived neurotrophic factor (GDNF) (8) and energy metabolites such as lactate for the development of testicular germ cells development (8, 9). One study reported that a combination of Sertoli cell culture-conditioned medium (SCM)+retinoic acid led to a differentiation of mesenchymal stem cells to germ-like cells (10).

In the present study, the focus shifted from the use of antioxidants to protect the testis, and instead, the researchers explored the effects of directly injecting SCM into the testis. The aim was to investigate whether this approach could promote the acceleration of testis recovery following MET treatment.

## 2. Material and Methods

### Animals, grouping, and sample collection

In this experimental study, 30 mature male Wistar rats (10–12 wk; 200 ± 10 gr) were purchased from the Pasteur Institute (Tehran, Iran) and randomly divided into 3 groups (n = 10) (11). The mature animals were adapted to new conditions and randomly divided into one control and 2 experimental groups, while newborns were only used for cell isolation. The animals had been kept in a standard condition (22 ± 2 C, 12 hr light/dark cycle), with ad libitum access to water and food.

Following the adaptation period, the animals were divided into control, MET (Sigma-Aldrich, MO), and MET+ SCM groups (n = 10/each). In the control group, animals did not receive any agent. In the MET group, animals received a single dose of MET (10 mg/kg intraperitoneally), once a week for 14 days (12). In the MET+SCM group, animals received MET with the same protocol. In addition, after 56 days (13, 14) on completing a spermatogenesis cycle, animals received SCM (10 µL), based on a previous study (11). Following 56 days, the animals were anesthetized and the serum, epididymis, and testicular samples were collected. The serum samples were used for hormonal analysis and stored at -80 C, the epididymis samples were used for sperm parameters examination, and testicular samples were fixed in 10% formalin fixative solution for further histopathological examinations. Finally, animals were humanely euthanized using a particular CO_2_ device (Tabriz-ADACO, Iran).

### Sertoli cells isolation and culture

In the current study, Sertoli cells were isolated from a 14-day-old newborn mice, as previously described (15). In brief, testicles were dissected out and moved to Dulbecco's modified Eagle's medium (DMEM; Cat: BI-1004, Bioidea, Iran). Then, they were de-capsulated and cut into small fragments. In continuation, the segments were suspended in DMEM medium, washed multiple times, and centrifuged for 1 min at 200 g. The seminiferous tubules following these steps were digested using collagenase type IV (2 mg/ml; Sigma-Aldrich, MO) for 20 min at 37 C. Next, DMEM was added to the digestion medium, washed 3 times, and after the last centrifugation, the cells were suspended in a DMEM medium. The cells were then passed through a nylon 100 mm mesh and cultured in a DMEM culture medium supplemented with 10% fetal bovine serum (Cat: 10437028, Gibco, USA) and pen-strip (0.25%; Cat: 03411, Bioidea, Iran) at 37 C 5% CO_2_ incubator. Following 48 hr, Sertoli cells were attached to the plates and the medium was changed to remove other possible residual germ cells from the culture condition. Moreover, free and non-adherent cells were removed by successively changing the culture medium.

### Sertoli cells purification approve, medium collection, and injection

**Figure 1:**
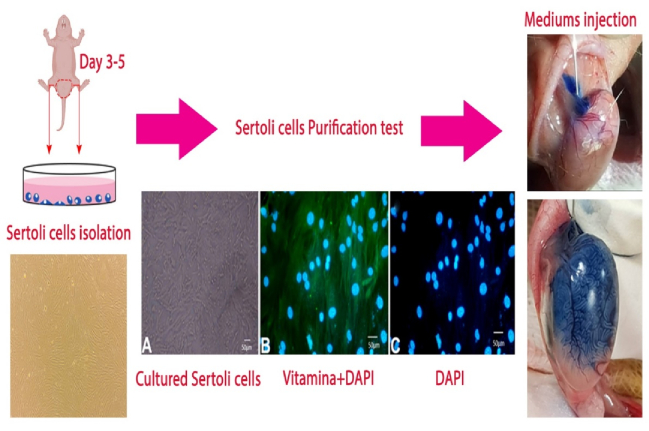
Flow chart of Sertoli cells isolation, culture, purification, and conditioned medium injection.

In week 1, vimentin protein (PA517229, Thermofisher Company, USA) was immunocytochemically examined to approve cell purification (Figure 1), as previously described (16). Sertoli cells were cultured and the cells' culture medium was removed, filtered every 2 days, and used as the injected SCM (12, 17). To inject SCM, animals were anesthetized using 5% ketamine (40 mg/kg) and 2% xylazine (18), and a 1 cm midline incision was made in the abdominal area. Then, the SCM was injected into both testicular tissues using an insulin syringe connected to a thin Pasteur pipette by a plastic tube. It should be noted that SCM was mixed with Trypan blue dye (at a ratio of 1:1; 100 µL; Cat: DB9733, DNABiotech, Iran) to confirm distribution (Figure 1). Finally, after confirmation, the testicles were returned to the scrotum. The incision was then closed using a nylon suture (4–0), and the animals were returned to their cages.

### Hormonal assessment

To examine luteinizing hormone (LH), follicular-stimulating hormone (FSH), and testosterone levels, collected serum samples were used. Serum levels of LH (Cat: 625–300), FSH (Cat: 425–300), and testosterone (3725–300) were evaluated by Monobind company kits (Monobind Inc., USA). The experiment process was based on the company's instructions and hormonal levels were calibrated according to the standard calibration curve of each hormone in the corresponding kit.

### Histopathological assessment

Almost 3–5 days' post-fixation, testicular tissues were routinely passaged and embedded in paraffin. Next, using an automatic rotary microtome, the blocks were cut (5–6 µm) and left to dry. The sections were stained with hematoxylin and eosin (H&E) and analyzed under a light microscope. In continuation, seminiferous tubules diameter (STD), epithelium thickness (ET), interstitial tissue diameter (ITD), tubular differentiation (TDI), spermiogenesis (SPI), and repopulation (RI) indices were examined. To analyze STD, ET, and ITD, ImageJ software (LOCI, Wisconsin, USA) was set and used to perform the measurements. The TDI, SPI, and RI were evaluated in randomly selected areas of sections (200 seminiferous tubules/animal was evaluated), as previously described (13).

### Sperm sampling, count, motility, and morphology

The epididymal tails were collected in a sterile condition, cut into various pieces, and moved into 1.5 ml microtubes of Ham's F10 (1 mL; Theromfisher, USA). To obtain the sperm, samples were incubated at 37.5 C and 5% CO_2_ for 30 min. Then, the epididymis tissues were removed from microtubes and a standard hemocytometer method was used for sperm count, motility, and progressive sperm motility, as previously described (19). Sperm samples were smeared on clean slides, left to dry, and fixed in an experimental grade of methanol (99%). Then, the slides were stained using H&E and analyzed under a light microscope ( × 100). It should be noted that at least 200 sperm per epididymis sample were analyzed.

### Ethical considerations

This study was conducted under the Veterinary Faculty of the Islamic Azad University, Tabriz, Iran “Animal Care and Use” Committee consideration and approval (Code: IR.IAU.TABRIZ.REC.1401.141).

### Statistical analysis

Both ICC and H&E slides were examined by an Olympus fluorescence and light microscope (Olympus, Japan), occupied with an onboard SONY camera (Zeiss, Cyber-Shot, Japan). To analyze sperm motility, a hot plate-occupied light microscope was used (Olympus, Japan). All images were designed using Adobe Photoshop CC version 2018 (Adobe System Inc., Mountain View, CA, USA). In the present study, all the findings were demonstrated as mean ± standard division (SD). The Kolmogorov-Smirnov statistic test was performed to normalize the quantitative data. One-way ANOVA and Dunnett's T3 multiple compression tests (GraphPad Prism version 9.0) were also performed to identify the statistical differences between groups. A p < 0.05 was considered statistically significant.

## 3. Results

### Hormonal examination

**Figure 2:**
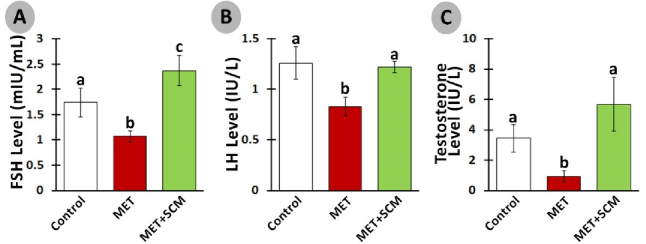
Effect of methotrexate (MET) and Sertoli cells conditioned medium (SCM) on follicle-stimulating hormone (FSH) (A), luteinizing hormone (LH) (B), and testosterone serum levels (C). All presented data are in Mean ± SD. Different superscripted letters represent significant differences (p ≤ 0.003, n = 10 rats in each group).

The serum level of LH was remarkably diminished (p ≤ 0.003) in the MET group vs. the control group. Meanwhile, SCM treatment increased (p < 0.003) LH level in the MET+SCM group when compared to the MET group (Figure 2A). Similarly, FSH and testosterone levels were significantly lower (p < 0.003) in the MET group animals compared to the control group rats. However, MET+SCM rats presented significantly higher (p < 0.003) FSH and testosterone levels vs. MET animals (Figures 2B and C).

### Histopathological evaluations

**Figure 3:**
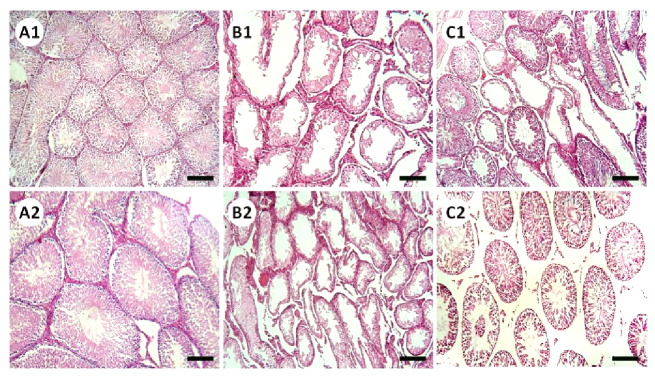
Cross sections of testicular tissues stained with H&E staining. Seminiferous tubules from the methotrexate (MET) group represent a deformed shape with arrested spermatogenesis and loss of germ cell integrity, while Sertoli cells conditioned medium (SCM) injected group ones demonstrate different aspects. Scale bar = 150 µm. (A1–2) Control group, (B1–2) MET group, (C1–2) MET+SCM group.

**Figure 4:**
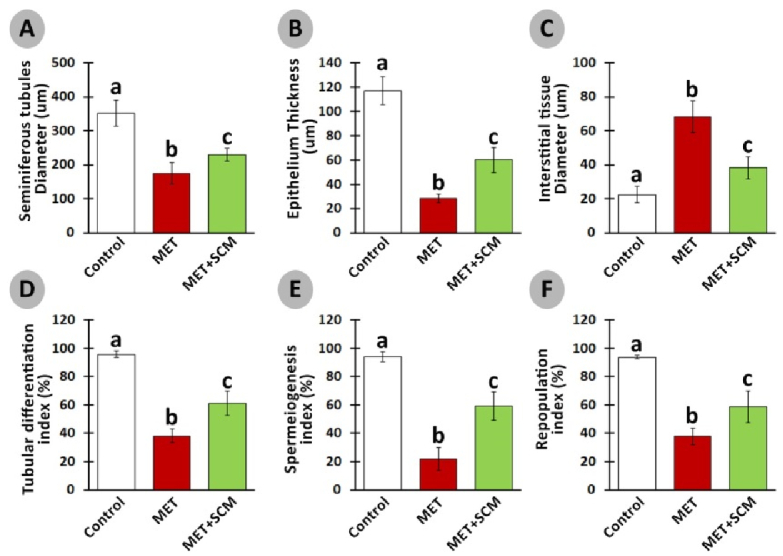
Effect of methotrexate (MET) and Sertoli cells conditioned medium (SCM) on seminiferous tubules diameter (A), epithelium thickness (B), interstitial tissue diameter (C), tubular differentiation (D), spermiogenesis (E), and repopulation indexes (F). All the data presented were in Mean ± SD. Different superscripted letters represent significant differences (p ≤ 0.002, n = 10 rats in each group).

Histopathological evaluation illustrated that MET animals demonstrated significant pathological defects, including depletion, dissociation of germ cells, the disintegration of seminiferous tubules' basal membrane, and spermatogenesis disruption in testicular tissue. However, SCM-received rats showed low pathological defects, whereas no pathological changes were observed in control animals. To examine pathological changes in the testicular tissues more precisely, STD, ET, ITD, TDI, SPI, and RI were evaluated, respectively (Figure 3). The results illustrated that MET significantly altered testicular histopathological parameters in MET animals compared to control rats. SCM treatment ameliorated histopathological parameters in MET+SCM animals. Moreover, our observations revealed that the MET+SCM group showed a remarkable (p ≤ 0.002) improvement in histopathological parameters when compared to the MET group (Figures 3 and 4).

### Spermatozoa count and viability

**Figure 5:**
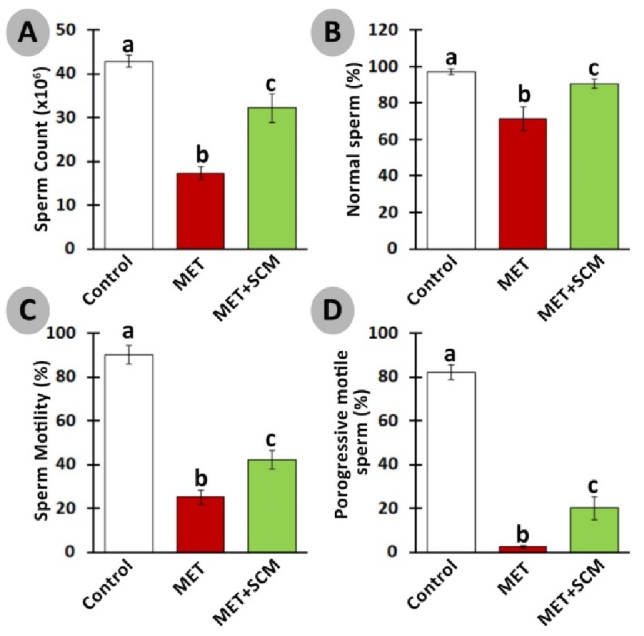
Effect of methotrexate (MET) and Sertoli cells conditioned medium (SCM) on sperm count (A), normal sperm (B), sperm motility (C), and progressive motile sperm (D). All the data presented are in Mean ± SD. Different superscripted letters represent significant differences (p ≤ 0.004, n = 10 rats in each group).

The rats in the met-induced group exhibited a remarkable (p < 0.003) decline in sperm count compared to the control group. Meanwhile, sperm count increased in MET+SCM animals vs. MET animals (Figure 5A). Similarly, morphologically normal, motile, and progressive sperm percentages significantly (p ≤ 0.004) diminished in the MET group vs. the control group. Meanwhile, SCM treatment could remarkably (p < 0.004) enhance these parameters in the SCM-treated group compared to the MET group (Figure 5B-D).

## 4. Discussion

Adverse effects of MET on male reproductive system have been reported in several studies (20, 21). Our results also showed this toxic agent led to severe damage to the testicular tissues, decreased serum levels of hormones including LH, FHS, and testosterone, and reduced sperm motility and count.

However, SCM administration could significantly ameliorate MET-induced alterations. Indeed, SCM injection enhanced LH, FSH, and testosterone levels remarkably in the MET+SCM group when compared to the non-treated MET group. It has been blatant that LH controls testosterone secretion from Leydig cells, while FSH stimulates Sertoli cells (21, 22). Moreover, a recently conducted study has illustrated that testosterone directly promotes Sertoli cells' GDNF production and secretion and GDNF-related receptor expression in SSCs (16). Diminished hormonal levels and disrupted hypothalamus-pituitary-testis axis interaction directly impact the spermatogenesis process and sperm production (23). Given that SCM injection increased LH, FSH, and testosterone levels, it can be suggested that SCM administration in post-MET treatment can positively improve hormonal recovery in rats. Similarly, one study reported that SCM administration increased LH and testosterone levels in busulfan-induced azoospermia mouse testis (11). In corroboration, it has been revealed that Sertoli cells mediate the testicular vascular network, leading to enhanced blood and hormone circulation in the testis (24). In addition, Sertoli cells can regulate the number and function of Leydig cells in interstitial tissue, leading to increase in the production of testosterone (25).

Furthermore, it has been revealed that MET administration in various doses, even low doses, adversely impacts STD, IND, spermatogenesis process, and testicular germ cell numbers (12, 20). Our observations demonstrated a significant reduction in STD, ET, ITD, TDI, SPI, and RI in MET-administrated animals compared to control animals, whereas SCM treatment could remarkably enhance all the mentioned parameters. Studies have reported that MET administration can generate high levels of reactive oxygen species, which in turn can cause adverse pathological changes in the testis (3, 26) and reduce intra-cytoplasmic carbohydrate and fatty acids accumulation, disrupting glucose-lactate production in Sertoli cells (27). It has been revealed that administration of Sertoli cells secreted stem cell factor had diminished testicular germ cell apoptosis (28). Furthermore, Sertoli cells are known to play a role in initiating the self-renewal and differentiation processes of SSCs by producing integrin- β and fibroblast growth factor-2; this mechanism ultimately contributes to the restoration of normal functioning in testicular tissues (29). One study reported a significant increment in the number of testicular germ/somatic cells and improved testicular histopathology post-SCM injection in busulfan-induced azoospermic mice (11). The SCM could positively enhance RI, TDI, and SPI in the SCM+MET group testis compared to the MET group. These findings suggest that SCM administration may improve testicular changes probably by providing germ cells with glucose, lactate, and growth factors.

Our results also showed a significant decrement in sperm count, motility, and morphology in the MET group compared to the control group, while a single injection of SCM could remarkably improve sperm parameters in the SCM+MET rats compared to the MET-non-treated rats. Previous studies conducted showed that MET by disrupting the hormonal balance in animals had negative impacts on sperm motility, morphology, and count (22, 26). It has been demonstrated that Sertoli cells functional disruption are associated with abnormal sperm morphology, motility, and count in mice (30). Like our findings, one study reported that SCM injection can increase the count and motility of sperms in mice with busulfan induced azoospermia. On the other hand, the ameliorative impact of SCM on hormonal balance and testicular tissue histopathology improvement may also be considered as effective parameters in improved sperm parameters.

## 5. Conclusion

It can be concluded that MET treatment led to hormonal imbalance, testicular damage, and decline in sperm parameters. However, a single dose SCM injection could significantly recover MET treatment-induced testicular pathological changes, FSH, LH, and testosterone serum levels, and sperm parameters. Improved testicular condition and FSH, LH, and testosterone levels may also indirectly lead to increased sperm count, increased motile and progressive sperm percentage, and sperms with normal morphology. Thus, it can be suggested that intra-testicular injection of SCM, even a single dose, can accelerate the recovery of the male reproductive system post-MET treatment. However, in order to fully understand the underlying mechanisms through which the SCM can improve the functional conditions of testicular tissue, further studies are necessary. These studies would help to elucidate the precise causes and mechanisms behind the beneficial effects of SCM on testicular tissues.

##  Data availability

The original data of the current study can be available from the corresponding and first authors upon reasonable request.

##  Author contributions

Masoud Delashoub had complete access to all the data in the study and takes responsibility for the integrity of the data and the accuracy of the data analysis. Concept and design were done by Masoud Delashoub. Acquisition and histopathological examination: Aref Delkhosh. Drafting of the manuscript and analysis of biochemical data: Fatemeh Shabani. Critical revision of the manuscript for important intellectual content: all authors. Statistical analysis: Masoud Delashoub. Supervision: Masoud Delashoub.

##  Conflict of Interest

The authors declare that there is no conflict of interest.
